# A Sphingosine-1-Phosphate Lyase Mutation Associated With Congenital Nephrotic Syndrome and Multiple Endocrinopathy

**DOI:** 10.3389/fped.2020.00151

**Published:** 2020-04-08

**Authors:** Avinaash Maharaj, Demetria Theodorou, Indraneel (Indi) Banerjee, Louise A. Metherell, Rathi Prasad, Dean Wallace

**Affiliations:** ^1^Centre for Endocrinology, John Vane Science Centre, William Harvey Research Institute, Queen Mary University of London, London, United Kingdom; ^2^Department of Paediatric Nephrology, Royal Manchester Children's Hospital, Manchester, United Kingdom; ^3^Department of Paediatric Endocrinology, Royal Manchester Children's Hospital, Manchester, United Kingdom

**Keywords:** *SGPL1*, sphingosine-1-phosphate lyase, congenital nephrotic syndrome, primary adrenal insufficiency, multiple endocrinopathy

## Abstract

**Background:** Loss of function mutations in *SGPL1* are associated with Sphingosine-1-phosphate lyase insufficiency syndrome, comprising steroid resistant nephrotic syndrome, and primary adrenal insufficiency (PAI) in the majority of cases. *SGPL1* encodes sphingosine-1-phosphate lyase (SGPL1) which is a major modulator of sphingolipid signaling.

**Case Presentation:** A Pakistani male infant presented at 5 months of age with failure to thrive, nephrotic syndrome, primary adrenal insufficiency, hypothyroidism, and hypogonadism. Other systemic manifestations included persistent lymphopenia, ichthyosis, and motor developmental delay. Aged 9 months, he progressed rapidly into end stage oligo-anuric renal failure and subsequently died. Sanger sequencing of the entire coding region of *SGPL1* revealed the novel association of a rare homozygous mutation (chr10:72619152, c.511A>G, p.N171D; MAF−1.701e-05) with the condition. Protein expression of the p.N171D mutant was markedly reduced compared to *SGPL1* wild type when overexpressed in an *SGPL1* knockout cell line, and associated with a severe clinical phenotype.

**Conclusions:** The case further highlights the emerging phenotype of patients with loss-of-function *SGPL1* mutations. Whilst nephrotic syndrome is a recognized feature of other disorders of sphingolipid metabolism, sphingosine-1-phosphate lyase insufficiency syndrome is unique amongst the sphingolipidoses in presenting with multiple endocrinopathies. Given the multi-systemic and progressive nature of this form of PAI/ nephrotic syndrome, a genetic diagnosis is crucial for optimal management and appropriate screening for comorbidities in these patients.

## Introduction

Sphingosine-1-phosphate lyase insufficiency syndrome (SPLIS) is a recently described condition, which in the main, incorporates steroid resistant nephrotic syndrome, and primary adrenal insufficiency (PAI). The disease is associated with loss of function mutations in *SGPL1*, encoding sphingosine-1-phosphate lyase that irreversibly binds sphingosine 1-phosphate (S1P) and commits it to the final degradative step in sphingolipid metabolism (1–3). Phenotypic expression of the condition is variable, from severe multi-systemic forms, associated with fetal hydrops and early mortality, to isolated single organ disease (1, 2, 4–9).

Nephropathy is associated with other genetic disorders of sphingolipid metabolism, such as Fabry and Gaucher disease, and sphingolipid accumulation is reported in glomerular disease of non-genetic origin (10). Endocrinopathy, including PAI, on the other hand is not commonly reported in the sphingolipidoses (11).

We report the novel association of a rare variant in *SGPL1* in a patient presenting with a severe combination of primary adrenal insufficiency and infantile onset nephrotic syndrome. This case further highlights the emerging multi-systemic phenotype in this rare disorder of sphingolipid metabolism, including its unique association with wider endocrinopathy.

## Case Presentation

A Pakistani male infant with 46XY karyotype, born to consanguineous parents, presented at 5 months of age with failure to thrive, metabolic acidosis and nephrotic syndrome, unmasked by a transient diarrhoeal illness. Postnatal growth failure was evident at presentation with a length of 58.5 cm (−3.05 SDS) and weight of 5.25 kg (−3.16 SDS) and a history of normal birthweight of 3.3 kg at 36 + 6 weeks gestation (+0.69 SDS). He was noted to have palmar hyperpigmentation, ichthyosis, under-virilized male genitalia and generalized developmental delay. Laboratory investigations revealed initially normal renal function, hypoalbuminaemia, mild lymphopenia, and nephrotic range albuminuria. There was an initial high anion gap metabolic acidosis which became normal anion gap acidosis following intravenous rehydration. Transient glycosuria and hypokalaemia prompted the consideration of proximal renal tubular acidosis, however these values normalized with no specific treatment other than rehydration and oral bicarbonate medication (Table 1). Ultrasound scanning demonstrated bilateral large, echogenic kidneys and calcification in both adrenals. The patient was treated with captopril, 1-alfacalcidol, sodium feredetate, ranitidine, bicarbonate 8.4% solution and energy-dense oral and nasogastric feeding. Oedema was relatively mild and managed with *as required* diuretic medication; 20% Human albumin infusions were not required.

**Table 1:** Results of laboratory investigations at initial (5 months) and last presentation (9 months).

**Test (reference range)**	**Acute presentation (district hospital)**	**At presentation (Tertiary hospital)**	**Last acute presentation (9 months old)**
UREA (2.5–6.0 mmol/L)	1.4	1	22.6
CREATININE (14–34 umol/L)	30	21	323
CYSTATIN C (0.7–1.5 mg/L)	N/A	1.52	5.26
SODIUM (133–146 mmol/L)	137	139	138
POTASSIUM (3.5–5.0 mmol/L)	3.3-4.5	4.5	6.2
BICARBONATE (19–28 mmol/L)	13	16	33
CHLORIDE (95–108 mmol/L)	109	113	87
eGFR BY CREATININE (ml/min/1.73*m*^2^)	N/A	107	8
eGFR BY CYSTATIN-C	N/A	48	15
CALCIUM (mmol/L)	2.31	2.11	1.51
PHOSPHATE (1.2–2.2 mmol/L)	1.97	1.79	2.74
PTH (1.6–6.9 pmol/L)	60	93.1	195.4
ALP (77–540 u/L)	524	505	198
ALBUMIN (28–40 g/L)	17	17	22
URINE PCR (0–30 mg/mmol)	>2,000	2,189	1,975
URINE ACR (0–3.0 mg/mmol)	N/A	1978.5	n/a
HEMOGLOBIN (100–130 g/L)	90	84	83
LYMPHOCYTES (3.3–11.5 × 10^9^/L)	0.9–1.6	1.52	0.58
PLATELETS (150–560 × 10^9^/L)	391	457	415
CHOLESTEROL (1.2–4.7 mmol/L)	N/A	4.8	7.6
ACTH (0–46 ng/L)^*^	999		
CORTISOL (133–537 nmol/l)^*^	61		
LH (1.7–8.6 IU/L), FSH (1.5–12.4 IU/L.)^*^	71, 27		
TSH (0.2–5 mu/L)^*^	27		
Free T4 (9–24 pmol/l)^*^	10.5		

* *Results prior to starting endocrine replacement therapy*.

The clinical phenotype and laboratory findings prompted early endocrine review and the additional diagnosis of PAI was made (low serum cortisol 61 nmol/L, markedly elevated ACTH 999 ng/L and low normal aldosterone 190 pmol/L). A wider endocrine phenotype included primary hypogonadism with microphallus and bilateral cryptorchidism (with raised basal gonadotrophins FSH 71 IU/L, LH 27 IU/L) and primary hypothyroidism (elevated TSH 25 mU/L and low fT4 10.7 pmol/L). Endocrine treatments included hydrocortisone, fludrocortisone and L-thyroxine replacement. Multiple hospital admissions ensued for nutritional and therapeutic interventions.

Renal function continued to deteriorate and the eGFR reduced from 107 mls/min/1.73m^2^ at 5 months to 31 mls/min/1.73m^2^ by 8 months (Chronic Kidney Disease (CKD) stage IIIb). Aged 9 months, the patient presented with acute oligo-anuric renal failure and pulmonary oedema following a brief diarrhoeal illness. Renal replacement therapy was declined by the family given the burden of morbidity to date and the patient subsequently died.

Initial genetic screening did not identify mutations within the standard SRNS genetics panel *(Bristol SRNS exome screen)*, noting only a likely non-pathogenic, heterozygous missense mutation in COL4A4 (NM_000092.4); Intron 41, c.3974-4dupT which was not consistent with phenotype. The unusual combination of nephrotic syndrome and primary adrenal insufficiency prompted consideration of the recently published multi-systemic syndrome of SGPL1 deficiency as a causative disease mechanism (1, 2).

## Materials and Methods

### Sanger Sequencing

*SGPL1* exons of interest (coding region) were amplified by PCR using specific primers as previously described (2).

### Sequence Interpretation and *in silico* Analysis of Variants

Using an autosomal recessive disease model, variants were assigned causality if they segregated within the proband family and were deemed pathogenic based on several *in silico* prediction software tools (SIFT; PolyPhen-2; Human Splicing Finder, version 3.0). Minor Allele Frequency was determined from the ExAC and Genome Aggregation Database (gnomAD) browsers.

### Protein Structure Modeling

An online tool, G23D (http://www.sheba-cancer.org.il/G23D) was used to map the genomic coordinates of pathogenic variant and predict functional alterations to mutant protein structure.

### *In vitro* Splicing Assay

An *in vitro* splicing assay was designed using the commercially obtained Exontrap cloning vector pET01 (MoBiTec GmbH, Göttingen, Germany) containing an intronic sequence interrupted by a multiple cloning site. DNA fragments of interest (insert length of 1,129 bp; exon 7 + 500 bp intronic sequence at both 5′ and 3′ ends) were amplified using a standard PCR protocol and specifically designed primers containing a restriction enzyme target site for XbaI. PCR products were sequenced, column purified using the QIAquick® PCR Purification Kit, and cloned into the Exontrap cloning vector pET01 (MoBiTec GmbH). The cloned sequences were verified by Sanger sequencing to ensure the fragment was in the correct orientation using pET01 specific primers. Wild-type (pET01-WT) and pET01 mutant plasmids were transfected into HEK293T cells using Lipofectamine 2000® reagent (Thermo Fisher Scientific). Total RNA, obtained from cells 24 h after transfection, was subjected to RT-PCR to generate cDNA with primer GATCCACGATGC (MoBiTec GmbH) and amplified with primers within the 5′ and 3′ exons in the pET01 vector. Amplification products were assessed on a 2% agarose gel.

### Site Directed Mutagenesis

Site directed mutagenesis of *SGPL1* Human Tagged ORF Clone (Origene) and oligonucleotide primer design was performed using the QuikChange II XL Site-Directed Mutagenesis Kit (Agilent) and web based QuikChange program in keeping with the manufacturer's guidelines.

### Western Blotting

CRISPR/ Cas9 engineered *SGPL1* knockout H295R cells were seeded into 6 well plates and transfected with wild type *SGPL1* and p.N171D mutant constructs using Lipofectamine 2000® reagent (Thermo Fisher Scientific). After 48 h whole cell lysates were prepared by addition of RIPA buffer (Sigma Aldrich) supplemented with protease and phosphatase inhibitor tablets (Roche). Protein concentrations were quantified using a Bradford protein assay (Bio-Rad). Lysates were denatured by addition of Laemmli sample buffer 2X (Sigma Aldrich) and boiled for 5 min at 98°C. 15–20 μg of protein were loaded into wells of a 4–20% SDS-PAGE gel (Novex) prior to electrophoretic separation using MOPS buffer. Protein transfer to PVDF membrane was achieved by electro-blotting at 15V for 45 min. The membrane was blocked with 5% fat free milk in TBS/0.1% Tween-20 and left to gently agitate for 1 h. Primary antibody (Human SGPL1 Antibody; AF5535, R&D Systems) was added at a concentration of 1:500 and β-actin (1:10,000) used as a housekeeping control. Primary antibody incubation was left overnight at 4°C with gentle agitation. The membrane was then washed for 5 min (X3) with Tris Buffered saline-Tween20 (TBST). Secondary anti-goat and anti-mouse antibodies were added at a concentration of 1:5,000 to blocking buffer and the membrane incubated at 37°C for 60–90 min. The membrane was subsequently washed three times (5 min each) with TBST and visualized with the LI-COR Image Studio software for immune-fluorescent detection.

### Study Approval

This study was approved by the Outer North East London Research Ethics Committee, reference number 09/H0701/12.

### Consent

Written informed consent was obtained from the parents or guardians of the participant for participation in the study and the publication of this case report.

## Results

**Figure 1:**
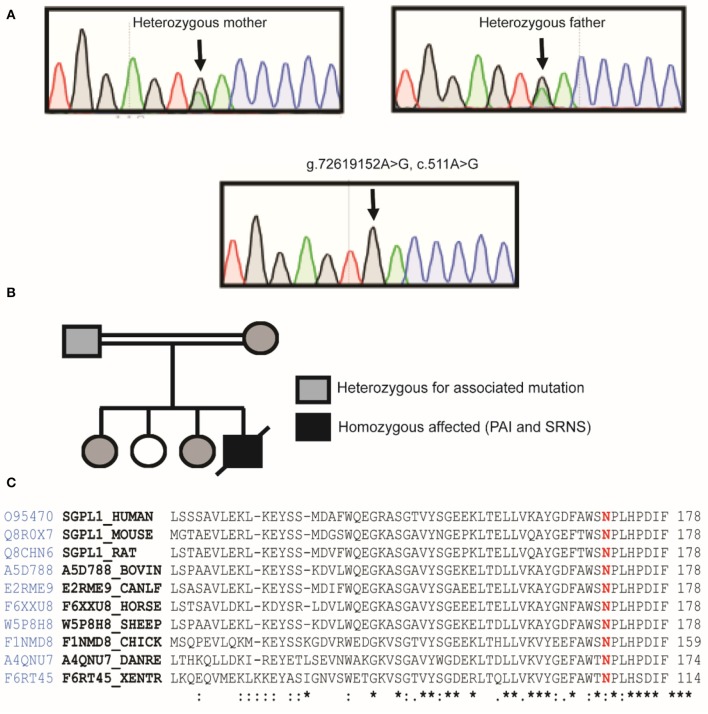
Our patient harbors a homozygous mutation in *SGPL1*, c.511A>G, p.N171D. **(A)** Partial sequence chromatograms of genomic DNA from the parents who are heterozygote carriers and the homozygote patient, showing the base change from A to G in exon 7. **(B)** Pedigree of affected patient. Black-filled symbols indicate individual homozygous, half-filled indicate individuals heterozygous for the mutation. White-filled symbols indicate wild-type individuals. **(C)** Partial alignment of SGPL1 protein sequences, showing conservation of asparagine (N) at position 171 across several species, highlighted in red, numbering relative to human sequence. Sequence conservation is beneath the alignment, *total conservation, : partial conservation.

**Figure 2:**
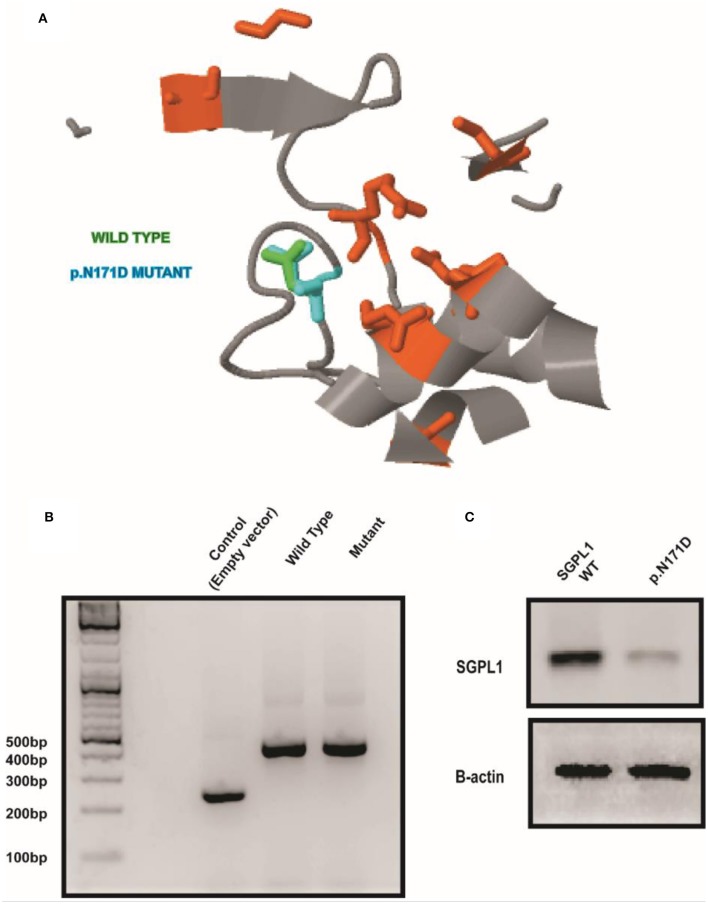
A conformational change in SGPL1 occurs with the p.N171D mutation, resulting in reduced protein expression. **(A)** Protein modeling showed a minor conformational change in SGPL1 due to amino acid substitution at position 171, predicted to result in reduced protein stability. **(B)** An *in vitro* splicing assay showed effective splicing of both wild type and mutant *SGPL1* exon 7 constructs providing no evidence of aberrant splicing. **(C)** Expression of p.N171D mutant in an *SGPL1* KO cell line is reduced when compared to wild type suggesting increased susceptibility to degradation.

Sanger sequencing of the entire coding region of *SGPL1* revealed a very rare homozygous missense mutation in *SGPL1* (chr10:72619152, c.511A>G; p.N171D) (gnoMAD MAF−1.701e-05; no homozygotes described) which was heterozygous in the unaffected consanguineous parents and siblings (Figures 1A,B). Partial alignment of SGPL1 protein sequences showed conservation of asparagine (N) at position 171 across several species (Figure 1C). Single nucleotide change c.511A>G (p.N171D), whilst denoted as deleterious across several platforms (SIFT, PolyPhen), showed minor conformational change on protein modeling that was likely to lead to protein instability but with a relatively low reliability score (Figure 2A). *In silico* prediction tools (Mutation Taster and Human Splicing Finder 3.0) suggested creation of an exonic splicing silencer site and aberrant splicing as the underlying pathogenic mechanism. An *in vitro* splicing assay revealed the mutant p.N171D heterologous mini-gene construct demonstrated unaltered splicing with normal incorporation of exon 7 between the constitutional exons of the exon trap vector with removal of the intervening sequences (Figure 2B). The p.N171D mutant plasmid generated by mutagenesis of an *SGPL1* Human ORF clone was expressed in a eukaryotic CRISPR/Cas9 engineered *SGPL1* KO H295R adrenocortical cell line and protein expression quantified by western blotting after 48 h. When compared to wild type, p.N171D was markedly reduced suggesting reduced stability and increased susceptibility to degradation in the mutant protein (Figure 2C).

## Discussion

*SGPL1* encodes sphingosine-1-phosphate lyase (SGPL1), which provides the single exit point for the sphingolipid metabolic pathway, irreversibly converting S1P to hexadecanal and phosphoethanolamine. Sphingosine-1-phosphate lyase insufficiency syndrome (SPLIS) describes the recently reported multi-systemic disease associated SGPL1 deficiency (clinical features summarized in Table 2) (1–9). Other mutations in the sphingolipid pathway are related to the sphingolipidoses, which include Gaucher, Fabry, and Niemann-Pick disease amongst others. Sphingolipids play an integral role as structural components of cell membranes, and sphingolipid intermediates such as S1P, sphingosine, and ceramide are signaling molecules involved in cell migration, differentiation, and cell survival.

**Table 2:** Summary of main clinical features of patients described with genetically confirmed sphingosine-1-phosphate lyase insufficiency syndrome (SPLIS) to date (1, 2, 4–9), including our own patient.

	**Patient numbers**	**Patient details**
Total patients	35 (+ 4 fetal demise)	Initial presentation at ages varying postnatally to 19 years
Sex (M:F)	21:14	
Deaths	15	Age at demise varying postnatally, with fetal hydrops, to 9 years; several precipitated by septic episodes
Nephrotic syndrome	29	Congenital to oldest presentation at 19 years with SRNS, with 19 patients presenting in infancy, six patients were reported to have renal transplantation (two of these required a second transplant).
Adrenal insufficiency	23^*^ (five additional cases with adrenal calcification on imaging)	Glucocorticoid deficiency in all cases ± mineralocorticoid deficiency. Age at presentation varying from early postnatal to oldest at 11 years, 11 patients presenting in infancy. 11 patients noted to have adrenal calcifications on imaging.
Hypothyroidism	12	Primary hypothyroidism with mildly raised TSH levels in all cases where biochemistry was reported
Gonadal dysfunction	7	Male patients presenting postnatally with cryptorchidism ± microphallus, all with raised gonadotrophins indicating primary gonadal failure.
Ichthyosis	12	
Neurological/ Developmental delay	18	Phenotypically heterogenous, varying from microcephaly, seizures, sensorineural deafness to later presentation with abnormal gait, peripheral neuropathy, progressive neurological deficit. Central and peripheral nervous system pathology variably reported. Neuroimaging findings varied from corpus callosum atrophy, cortical atrophy, cerebellar hypoplasia to basal ganglia involvement.
Immunodeficiency	13	Most consistently this is an absolute lymphopenia, some patients additionally with hypogammaglobulinemia and neutropenia.
Other features	<5 patients	Dysmorphic features, skeletal abnormalities, liver dysfunction are reported.

* *Indicates biochemically proven*.

SGPL1 deficiency leads to an accumulation of upstream bioactive sphingolipids, often in a tissue-specific manner (1, 2, 9). SGPL1 is found in the endoplasmic reticulum of renal podocytes, mesangial, and endothelial cells and immunofluorescence studies show co-localization in the renal proximal tubules, which may explain the initial biochemical picture in our patient consistent with proximal renal tubular dysfunction (1). Furthermore, it has been hypothesized that S1P accumulation leads to a signaling defect of angiogenic factors, resulting in additional changes within the renal parenchyma (6). The enzyme is moderately expressed in the adrenal cortex, testes and thyroid gland (2); the endocrine organs affected in our patient. The sphingolipid intermediates have been implicated in the regulation of acute steroidogenesis and expression of steroid responsive transcriptional elements while SGPL1 deficient mice have abnormal adrenal and testicular morphology (2, 12, 13), in keeping with the phenotype seen in the human disease.

We present a novel association of a rare variant within *SGPL1* associated with a severe phenotype of SGPL1 deficiency, encompassing infantile-onset nephrotic syndrome with a wider endocrine phenotype including PAI, primary hypothyroidism and primary hypogonadism. In addition, our patient also demonstrated persistent mild lymphopenia, ichthyosis, and neurodevelopmental delay, features similar to previously described cases.

To date, there have been 35 published genetically confirmed cases involving 20 kindreds and 20 different mutations in *SGPL1* (including this patient, not including those with fetal demise) (1, 2, 4–9) (see Tables 2, 3). Patients present with congenital or steroid resistant nephrotic syndrome (see Tables 2, 3) and progress to end-stage renal disease with renal biopsy histological findings of focal segmental glomerulosclerosis and diffuse mesangial sclerosis. Similarly, the majority of affected individuals have adrenal disease (see Tables 2, 3). SGPL1 has a potential role to play in other endocrine tissues with emerging reports of wider endocrinopathy (thyroid and testicular), as seen in our patient (1, 2, 5–9). This appears to be unique to SPLIS in comparison to other sphingolipidoses. In common with several disorders of sphingolipid metabolism, ichthyosis is reported (1, 2, 9). Neurological dysfunction can affect both the central and peripheral nervous system and is described with progressive morbidity. Interestingly, mutations inherited in compound heterozygosity have been associated with isolated peripheral neuropathy akin to Charcot-Marie Tooth; the adult siblings described do not have additional disease (4). Lymphopenia is seen in some patients and is associated variably with an increased frequency of infections (1, 2, 5, 9). SPLIS therefore encompasses a clinically heterogenous phenotype, with the most severe cases presenting with fetal hydrops, whilst others are affected with single organ disease alone.

**Table 3:** Phenotypic characterization of mutations in *SGPL1*.

**Mutation**	**Deaths**	**SRNS**	**PAI**	**Hypothyroidism**	**Gonadal dysfunction**	**Ichthyosis**	**Neurological features**	**Immunodeficiency**
p.S3Kfs*11	1/4	+	+	+	–	+	±	±
c.261+1G>A	–	+	+	+	+	+	+	+
p.E132G^¥^	–	+	+	–	–	+	–	–
p.N171D	1/1	+	+	+	+	+	+	+
p.I184T^†^	–	–	–	–	–	–	+	–
p.S202L^Ψ^	–	+	–	+	–	–	+	+
p.R222W	2/2	+	+	–	–	–	±	±
p.R222Q	1/9	+	+	–	–	–	+	±
p.R278Gfs*17^¥^	–	+	+	–	–	+	–	–
p.F290L^δ^	1/1	+	+	+	+	+	+	+
p.L312Ffs*30	–	+	+	–	+	–	–	–
p.A316T^Ψ^	–	+	–	+	–	–	+	+
p.Y331*^δ^	1/1	+	+	+	+	+	+	+
p.R340W	2/2	+	+	+	–	–	–	–
p.S346I	3/3	+	+	+	–	+	+	+
p.S361*^†^	–	–	–	–	–	–	+	–
p.F411Lfs*56	1/1	+	+	+	+	–	+	+
p.Y416C	–	+	+	+	–	–	+	+
p.R505*	2/2	+	+	–	+	–	–	–
p.F545del	1/1	+	+	+	–	+	+	+

¥†Ψδ denotes compound heterozygous mutations.

Sources of published mutations: Lovric et al. (1), Prasad et al. (2); Atkinson et al. (4); Bamborschke et al. (5); Janecke et al. (6); Linhares et al. (7); Settas et al. (8); Taylor et al. (9).

*Our patient's mutation is denoted in red. + denotes presence in all; ± denotes presence in some but not all patients; – denotes absence of clinical feature. Deaths reported all occurred at <10 years of age*.

**Figure 3:**
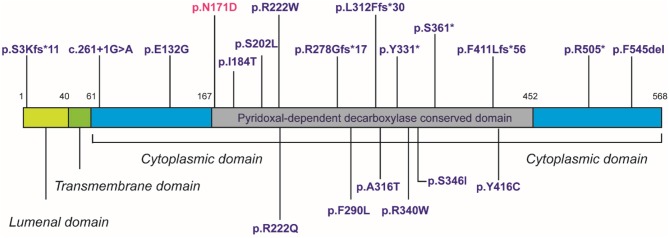
Domain topology showing distribution of 20 published mutations.

Despite *in silico* predictions, the p.N171D *SGPL1* mutant showed unaltered splicing in comparison to wild-type, however protein expression was markedly reduced as was anticipated by the instability predicted by protein modeling. Whilst all functional characterizations of *SGPL1* variants, described in this condition so far, demonstrate markedly reduced protein expression, clinical severity has not always correlated (1, 2). It has been proposed that individuals with most severe phenotypes harbor mutations within the active pyridoxal-dependent decarboxylase domain (5), which is home to the variant in our presented case (Table 3, Figure 3). However, milder phenotypes characterized solely by axonal polyneuropathy (Charcot Marie Tooth) due to mutations p.S361^*^ and p.I184T also occur within this region. As yet, there is no evident genotype- phenotype correlation in SPLIS, with equally severe clinical phenotypes seen in mutations outside the active region and in both frameshift and missense mutations (Table 3, Figure 3). Phenotypic heterogeneity is also evident in individuals with the same *SGPL1* variants, even within kindreds. Further investigation of the genotype together with detailed clinical phenotyping (at presentation and longitudinally) is required, as more individuals are diagnosed. Establishing a genotype/ phenotype correlation is further complicated by the consanguinity of the affected kindreds and the likely presence of other genetic modifiers of disease.

The recent description of SPLIS finally provides a diagnostic link for patients presenting with congenital NS and PAI. In view of the reports of isolated adrenal or renal disease at presentation it would be pertinent for *SGPL1* to be considered in diagnostic genetic panels for PAI/SRNS and certainly if seen in combination. Given the phenotypic variability of this disease, the genetic diagnosis may be crucial for confirming the clinical suspicion and facilitating initiation of appropriate screening and treatment for patients. Importantly, early consideration of this disease will hasten identification of associated co-morbidities. This could, for instance, reduce the rate of CKD progression and minimize the long-term complications of the associated endocrinopathy. The published literature suggests that the prognosis for these patients is poor with just under 50% mortality (15/35 reported cases, with 4 additional cases of fetal demise), the majority of those deaths in the first year of life. Future research will potentially lead to targeted genetic therapies but for now, a heightened awareness of the syndrome will allow earlier recognition, reducing the time to diagnosis and appropriate intervention and inform genetic counseling for affected families.

## Data Availability Statement

The raw data supporting the conclusions of this article will be made available by the authors, without undue reservation, to any qualified researcher.

## Ethics Statement

The studies involving human participants were reviewed and approved by the Outer North East London Research Ethics Committee, reference number 09/H0701/12. Written informed consent to participate in this study was provided by the participants' legal guardian/next of kin. Written informed consent was obtained from the individual(s), and minor(s)' legal guardian/next of kin, for the publication of any potentially identifiable images or data included in this article.

## Author Contributions

DW, DT, and IB recruited and clinically characterized patient. AM, RP, and LM conducted Sanger sequencing and analysis of data. AM conducted protein modeling and functional analysis of the *SGPL1* mutant described. DW, AM, and RP prepared the draft manuscript. All authors contributed to the discussion of results, edited, and approved the final manuscript.

### Conflict of Interest

The authors declare that the research was conducted in the absence of any commercial or financial relationships that could be construed as a potential conflict of interest.
